# Is hypermetropia regressing in accommodative esotropia?

**DOI:** 10.5935/0004-2749.20210020

**Published:** 2025-02-02

**Authors:** Fatma Yulek, Sema Yuzbasioglu, Demet Eyidogan

**Affiliations:** 1 Ophthalmology Department, Ankara Atatürk Education and Research Hospital, Ankara Yıldırım Beyazıt University Medical Faculty, Ankara, Turkey

**Keywords:** Amblyopia, Accommodation, ocular, Esotropia, Refraction, ocular, Ambliopia, Acomodação ocular, Esotropia, Refração ocular

## Abstract

**Purposes:**

To evaluate the relationship of changes in hypermetropia and ocular alignment in patients with accommodative esotropia.

**Methods:**

The medical records of consecutive patients diagnosed with refractive accommodative esotropia (esotropia eliminated or decreased to within 10 D with full hyperopic correction) were retrospectively reviewed. Cycloplegic refractions culled from medical records were converted into spherical equivalents. Presence of amblyopia, changes in refractive error and ocular alignment at admission and after the follow-up period were evaluated.

**Results:**

Seventy patients (mean age: 6.01 ± 5.41 years; female: 60.6%; mean follow-up: 5.8 ± 3 years) had corrected esotropia of 40 ± 20 prismatic diopters at admission. The average decrease per year in near and distance deviations with glasses was 1.71 ± 3.96 prismatic diopters/year and 1.09 ± 3.25 prismatic diopters/ year, respectively. The total myopic shift of the right and left eyes was 1.08 ± 1.35 D and 1.20 ± 1.40 D, respectively. Myopic shift/year was 0.22 D/year and 0.26 D/year, respectively. The correlation between the rate of myopic shift and rate of change in corrected near deviation was weak. The correlation for the rate of myopic shift was not high for the right and left eyes (r=0.18; p=0.15).

**Conclusion:**

The amount of deviation and hypermetropia gradually decreased in accommodative esotropia during follow-up. On the other hand, it may be incorrect to assure patients that the amount of deviation will decrease in parallel with the refractive error.

## Abstract (pt)

**Objetivo:**

Avaliar a relação entre alterações na hipermetropia e o alinhamento ocular em pacientes com esotropia acomodativa.

**Métodos:**

Foram analisados retrospectivamente prontuários médicos de pacientes consecutivos diagnosticados com esotropia acomodativa refrativa (com esotropia eliminada ou reduzida a menos de 10 D com correção completa da hiper metropia). As medidas de refração em cicloplegia obtidas dos prontuários foram convertidas em equivalentes esféricos. Avaliaram-se ainda a presença de ambliopia, alterações do erro refrativo e o alinhamento ocular à admissão e depois do período de acompanhamento.

**Resultados:**

Setenta pacientes (média de idade=6,01 ± 5,41 anos, 60,6% do sexo feminino, acompanhamento médio de 5,8 ± 3 anos) apresentaram esotropia de 40 ± 20 dioptrias prismáticas (DP) para perto à admissão. A diminuição média anual no desvio para perto e para longe com o uso de óculos foi de 1,71 ± 3,96 DP/ano e 1,09 ± 3,25 DP/ano, respectivamente. Os desvios miópicos totais dos olhos direito e esquerdo foram de 1,08 ± 1,35 D e 1,20 ± 1,40 D, respectivamente. Os desvios miópicos anuais foram de 0,22 D/ano e 0,26 D/ano para os olhos direito e esquerdo, respectivamente. A correlação entre a taxa de desvio miópico e a taxa de alteração do desvio para perto corrigido foi fraca. A correlação da taxa de desvio miópico não foi alta para os olhos direito e esquerdo (r=0,18, p=0,15).

**Conclusão:**

A quantidade de desvio e a hipermetropia diminuem gradualmente na esotropia acomodativa durante o acompanhamento. Por outro lado, pode não ser apropriado garantir aos pacientes que o desvio diminuirá em paralelo ao erro refrativo.

## INTRODUCTION

Refractive accommodative esotropia is frequently associated with moderate or high hypermetropia^(1)^. Treat ment of cycloplegic refractive error is the mainstay of therapy for the prevention of amblyopia and maintenance of strereopsis^(2)^. We frequently encounter questions from the families of these patients regarding the duration of spectacle wear and possibility of refractive changes with time. The abnormal accommodation reflex required to overcome blurring caused by undercorrection of hypermetropia may still persist after myopic shift in some patients^(3)^. Some patients may continue to use spectacles for maintenance of good ocular alignment, despite a decrease in their hypermetropia. On the other hand, some patients may require spectacles for better vision to correct the hypermetropia that has not regressed. This retrospective study was designed to evaluate the patterns of change in ocular alignment and refractive error in patients with refractive accommodative esotropia during follow-up.

## METHODS

The protocol for this research project was approved by the Ethics Committee of our institution within which the work was undertaken, and conforms to the tenets of the Declaration of Helsinki in 1995 (as revised in Edinburgh in 2000). The medical records of 70 patients with refractive accommodative esotropia, who attended the Pediatric Ophthalmology Clinic between 2005 and 2017, were reviewed. The best corrected visual acuity, cycloplegic refraction, degree of distance and near deviations with and without glasses, presence of amblyopia (diagnosed by a decrease in best corrected visual acuity not attributable to any clinically detected structural abnormality of the eye with an underlying amblyogenic factor, including strabismus, anisometropia [defined as ≥1.5 D difference in spherical equivalent or cylinder]), were recorded at the initial and final visits. Patients with hypermetropia >2 D whose esodeviation was corrected with glasses were included. The exclusion criteria were: presence of neurological problems; mental and motor retardation; other ocular pathologies; comitant vertical strabismus; and incomitant strabismus. All patients underwent visual acuity measurement using the Snellen chart, cycloplegic refraction with 1% cyclopentolate, ophthalmological examination (including orthoptic and biomicroscopic examination), Worth’s four-dot test, and TNO stereoacuity test (if cooperative). Spherical equivalent was calculated by summing the spherical error with half of the cylindrical error. The myopic shift for each eye was calculated by subtracting the final refractive error from the initial refractive error. In cooperative children, the deviation was measured at 33 cm and 6 m with prisms and an alternate cover test. The Krimsky test was used in uncooperative children. The cyclopegic refraction and deviations were evaluated every 3-6 months.

The Kolmogorov-Smirnov test was used to evaluate the normal distribution of data. The preand posttreatment values were compared by a paired t-test, and differences between patients with refractory amblyopia and those without amblyopia was evaluated using Student’s t-test. The p-values <0.05 denoted statistically significant differences.

## RESULTS

The mean follow-up period for the 70 patients was 70 ± 36 months (mean age at admission: 6.01 ± 5.41 years; females: 60.6%). The means of best corrected visual acuity (logMAR), spherical refractive error of each eye, as well as distance and near deviations with glasses at admission and final visit are depicted in table 1. During the follow-up, bifocals (11.4%, n=8) and surgery (20%, n=20) were used in addition to full cycloplegic refractive correction to control the deviation.

**Table 1:** The means of best corrected visual acuity (logMAR), spherical refractive error of each eye, and distance and near deviations with glasses of the patients at admission and final visit

	Admission		Final visit		p-value
	Right	Left	Right	Left	
BCVA	0.17 ± 0.19	0.19 ± 0.22	0.04 ± 0.06	0.08 ± 0.181	0.001
Mean spherical refractive error	+4.20 ± 2.06	+4.65 ± 2.22	+2.94 ± 1.77	+ 3.32 ± 1.88	0.001
Near deviation with glasses	17 ± 10PD		6 ± 6PD		0.001
Distance deviation with glasses	7 ± 6PD		2 ± 3 PD		0.01

BCVA= best corrected visual acuity; PD= prism diopter.

At the final visit, the spherical equivalent (p=0.001), near (p=0.001), and distance (p=0.011) deviations with glasses were significantly decreased compared with the initial values obtained at admission.

The mean total myopic shift was 1.08 ± 1.35 D and 1.20 ± 1.40 D for the right and left eyes, respectively. During the mean follow-up of 5.83 years, the myopic shift/year was 0.22 D/year and 0.26 D/year for the right and left eyes, respectively.

Although the correlation of total myopic shift of the right and left eyes was high (r=0.77; p<0.0001) the correlation for their rate of myopic shift was weak (r=0.18; p=0.15). The rate of change in near and distance deviations with glasses was 1.71 ± 3.96 PD/year and 1.09 ± 3.25 PD/year, respectively. The correlation of the rate of change in near and distance deviations was strong (r=0.90; p<0.0001).

The rate of change in distance and near deviations was weakly correlated with the rate of myopic shift of the right and left eyes (Table 2).

**Table 2:** The correlation of the rate of myopic shift of the right and left eyes with the rate of change in distance and near deviation with glasses

		Mean rate of myopic shift of right eye (D/year)	Mean rate of myopic shift of left eye (D/year)
Rate of change in near deviation (PD/year)	r	-0.17	0.08
	p-value	0.15	0.52
Rate of change in distance deviation (PD/year)	r	-0.15	0.09
	p-value	0.23	0.48

D= diopter; PD= prism diopter.

Amblyopia was detected in 24% of patients, while 50% of the patients did not have amblyopia at admission. All patients with amblyopia had treatment involving refractive correction and patching. At the final visit, 23% of the patients had refractory amblyopia. The total myopic shift, and change in distance and near deviations were not significantly different between patients with refractory amblyopia and those without amblyopia at the final visit (p>0.05) (Table 3). The rate of myopic shift and rate of change in distance and near deviations were also not significantly different between these groups (p>0.05) (Table 4).

**Table 3:** Myopic shift, and change in near and distance deviations in patients with refractory amblyopia and those without amblyopia

	Patients without amblyopia		Patients with refractory amblyopia		p-value
	Mean	SD	Mean	SD	
Myopic shift of the right eye (D)	1.03	1.36	1.05	0.73	0.96
Myopic shift of the left eye (D)	1.12	1.28	1.11	1.09	0.97
Change in near deviation (PD)	6.47	10.68	7.06	13.06	0.85
Change in distance deviation (PD)	3.20	7.57	3.69	8.21	0.82

SD= standard deviation; D= diopter; PD= prism diopter.

**Table 4:** Rate of myopic shift and rate of change in near and distance deviations in patients with refractory amblyopia and those without amblyopia

	Patients without amblyopia		Patients with refractory amblyopia		p-value
	Mean	SD	Mean	SD	
Rate of myopic shift of the right eye (D/year)	0.20	0.40	0.17	0.14	0.78
Rate of myopic shift of the left eye (D/year)	0.26	0.51	0.24	0.23	0.82
Rate of change in near deviation (PD/year)	1.67	3.87	1.98	4.68	0.79
Rate of change in distance deviation (PD/year)	1.11	3.41	1.22	3.12	0.91

SD= standard deviation; D= diopter; PD= prism diopter.

## DISCUSSION

Prompt treatment of deviation in intermittent and continuous esotropia is crucial for improving motor and binocular function^(2)^. It has been shown that fair and poor compliance with spectacle use greatly increases the risk of poor sensory and motor outcomes in children with pure refractive accommodative esotropia^(1)^. Thus, all these patients had full cycloplegic correction to control esodeviation. On the other hand, controlled studies could not be performed in this patient group who had full cycloplegic correction in early stages. Consequently, families usually experience anxiety and have questions related to the improvement of hypermetropia, as well as deviation.

In this study, early changes in deviation and refraction in this patient group with spectacles were evalua ted. The mean hypermetropia at admission (4.20 ± 2.06 D for the right eye and +4.65 ± 2.22 D for the left eye; mean age: 6.01 ± 5.41 years) was relatively low com pared with that measured in other studies involving younger patients (+5.03 ± 1.87 D and +5.12 ± 1.54 D for the right and left eyes, respectively)^(4)^. After a follow-up of 70 ± 36 months, the mean spherical equivalent was decreased to +3.12 ± 1.82 D and +3.28 ± 1.89 D for the right and left eyes, respectively. At near, the esodeviation with glasses was decreased at a mean rate of 1.71 ± 3.96 PD/year; at distance, this rate was 1.09 ± 3.25 PD/year. The myopic shift/year for the right and left eyes was 0.22 D/year and 0.26 D/year, respectively.

Birch et al. reported a spherical equivalent of +5.67 ± 1.26 D in the late-onset accommodative esotropia group, in whom deviation was initiated at 18-48 months of age^(5)^. They could not observe myopic shift in the lateonset group; however, they reported a myopic shift of 0.43 D/year in patients with early-onset accommodative esotropia that was initiated at <1 year of age. In their study, the total myopic shift in the early-onset group was 1.45 ± 2.16 D.

The population of our study was similar to the lateonset group of this previous investigation in terms of age distribution, and had a myopic shift/year of 0.22 D/year and 0.26 D/year for the right and left eyes, respectively. The follow-up period was longer in the previous study and the initial hypermetropia was higher (patients with moderate and high hypermetropia were included). The lower rate of myopic shift observed in our study compared with that noted by Birch et al. may be explained by the lower initial hypermetropia recorded in our patients. Notably, there are also studies reporting continuous myopic shift^(6)^.

Birch et al. suggested that, in patients with anisometropia, highly hypermetropic eyes have more myopic shift, while amblyopic eyes exhibit a slower rate of myopic shift^(5)^. On the other hand, Park et al. reported that amblyopic eyes showed greater decreases in spherical equivalent compared with nonamblyopic eyes. In addition, most patients in the hyperopic group showed a great decrease in hyperopia over time, supporting our findings. We could not find a significant difference between patients with and without amblyopia in terms of total myopic shift, distance and near deviations, and their rate of change (p>0.05) (Tables 3 and 4). Our follow-up period was shorter and the differences in populations may explain the different observations versus the study conducted by Birch et al.^(5)^.

Park et al. reported a mean annual decrease in spherical equivalent (during a 2-year follow-up period) of 0.30 D/y for the right eye and 0.40 D/y for the left eye^(7)^. Some previous studies of Western cohorts reported that hyperopia remained unchanged or increased until 5-10 years of age and decreased thereafter^(8-11)^.

Several studies proposed that refractive correction in esotropia prevents emmetropization^(12,13)^. Köse et al. reported that full or partial correction of refractive error in accommodative esotropia does not affect the final refractive status^(14)^. The amount of emmetropization was relatively low in the study conducted by Köse et al. (in the partial correction group from 5.29 D to 4.9 D). On the contrary, Hutcheson et al. proposed that the baseline and final refractive errors were significantly lower in the children successfully weaned from spectacles (p=0.014). Of note, the children who were successfully weaned from spectacles were older at the time of initial diagnosis with accommodative esotropia (4.6 vs. 2.5 years)^(15)^, inconsistent with the results reported by Birch et al.^(5)^.

Consequently, the dilemma of emmetropization and the effect of early correction of full cycloplegic correction persists in this patient group. However, significant change in deviation is obvious. Decreasing the amount of deviation is important for motor and sensorial development^(2)^.

Another finding in our study is the low correlation of the rate of change in hypermetropia of the right and left eyes. This may imply that we may need to decrease hypermetropia in different degrees in each eye. However, this approach is usually not practical in clinical practice.

There were several limitations in this study. All patients with accommodative esotropia, including partially refractive accommodative esotropia (since some patients required surgery in addition to refractive correction), were included. The number of patients with different types of accommodative esotropia can be increased to evaluate changes in each type. Since most patients had low and moderate hypermetropia, the results cannot be extrapolated to those with high hypermetropia. Furthermore, we did not include patients with early-onset (<1 year of age) accommodative esotropia. Several factors, such as age at which the use of spectacles was initiated and the decreasing rate of hypermetropic correction, may influence the final results which should be evaluated in prospective studies.

Finally, it is important to inform families that refraction may decrease in these patients with accommodative esotropia and low-to-moderate hypermetropia at the initial visit, although to a lesser degree compared with near deviation. It is also important to independently evaluate each eye for reductions in the degree of hypermetropia as the treatment evolves and decreases in deviation since their rates do not appear to be correlated.
